# Rotigotine suppresses sleep-related muscle activity augmented by injection of dialysis patients’ sera in a mouse model of restless legs syndrome

**DOI:** 10.1038/s41598-019-52735-z

**Published:** 2019-11-08

**Authors:** Kazuhiro Muramatsu, Sachiko Chikahisa, Noriyuki Shimizu, Hiroyoshi Séi, Yuichi Inoue

**Affiliations:** 1 Department of Pediatrics, Jichi Medical University, Tochigi, Japan; 2 Department of Pediatrics, Gunma University Graduate School of Medicine, Gunma, Japan; 3 Department of Integrative Physiology, Institute of Biomedical Sciences, Tokushima University Graduate School, Tokushima, Japan; 4 Department of Somnology, Tokyo Medical University, Tokyo, Japan

**Keywords:** Neurophysiology, Sleep disorders

## Abstract

Idiopathic restless legs syndrome (RLS) has a genetic basis wherein *BTBD9* is associated with a higher risk of RLS. Hemodialysis patients also exhibit higher rates of RLS compared with the healthy population. However, little is known about the relationship of *BTBD9* and end-stage renal disease to RLS pathophysiology. Here we evaluated sleep and leg muscle activity of *Btbd9* mutant (MT) mice after administration of serum from patients with either idiopathic or RLS due to end-stage renal disease (renal RLS) and investigated the efficacy of treatment with the dopamine agonist rotigotine. At baseline, the amount of rapid eye movement (REM) sleep was decreased and leg muscle activity during non-REM (NREM) sleep was increased in MT mice compared to wild-type (WT) mice. Wake-promoting effects of rotigotine were attenuated by injection of serum from RLS patients in both WT and MT mice. Leg muscle activity during NREM sleep was increased only in MT mice injected with serum from RLS patients of ideiopatic and renal RLS. Subsequent treatment with rotigotine ameliorated this altered leg muscle activity. Together these results support previous reports showing a relationship between the Btbd9/dopamine system and RLS, and elucidate in part the pathophysiology of RLS.

## Introduction

Restless legs syndrome (RLS) is a common sensorimotor disorder, whose basic components include a sensory experience, akathisia, and a sleep-related motor sign, periodic leg movements during sleep (PLMS), both associated with an enhancement of the individual’s arousal state^1^. Although the detailed pathological mechanisms of RLS are unclear, RLS occurs in an idiopathic form of genetic origin. Variants in *BTBD9*, *MEIS1*, *MAP2K5/SKOR1*, and *PTPRD* are known to confer a significant risk for RLS^2–5^. The most common conditions associated with RLS include iron deficiency, renal failure, neuropathy, spinal cord pathology, pregnancy, and multiple sclerosis. The use of certain medications, including selective serotonin reuptake inhibitors, lithium, dopamine antagonists, and caffeine can also increase the rate of RLS^6–8^. Patients with end stage renal disease (ESRD) receiving hemodialysis also have a remarkably higher incidence of RLS relative to the healthy population^9,10^.

Non-medication therapies such as iron supplementation and life guidance and drug therapies involving dopamine agonists have been widely used to treat RLS. Dopamine therapies have also been reported to be effective in treating RLS symptoms associated with chronic kidney disease^9,11,12^. Rotigotine, a non-ergot dopamine agonist that stimulates all dopamine receptor subtype (D_1_-D_5_), is approved for the symptomatic treatment of moderate to severe idiopathic RLS^13^. In a rat model of RLS caused by lesioning of dopaminergic nuclei, exposure to the neurotoxin 6-hydroxydopamine (6-OHDA) prevented increases in the frequency of limb movement^14^, whereas treatment with the dopaminergic agonist pramipexole decreased limb movement frequency^14^, suggesting that the dopamine system is involved in pathological mechanisms associated with RLS.

*BTBD9* encodes a protein that has two highly conversed domains, the BTB/POZ domain and the BACK domain, which are both associated with transcriptional regulation and protein ubiquitination. In humans, a genome-wide association (GWAS) study indicated that individuals carrying *BTBD9* variants have increased risk of familial RLS^3^. *BTBD9* variants are also associated with RLS in ESRD^15^, although another study reported differing results^9^. In animal models of RLS, *Btbd9* mutant mice exhibited an increased travel distance in an open field test and an increased amount of voluntary activity in a wheel running analysis during the rest phase; these changes were accompanied by altered serum iron levels^16^. In *Drosophila*, loss of the *BTBD9* homolog CG1826 (dBTBD9) resulted in sleep fragmentation and increased motor activity^17^. The same report indicated that BTBD9 controls dopamine levels in fly brains and iron homeostasis in human cell lines^17^. However, little is known about the physiological function of *BTBD9* and its relationship to RLS pathophysiology^18,19^. In particular, how *BTBD9* is associated with RLS that frequently occurs in hemodialysis patients is unclear. We also used a mouse model to examine how genetic mutations such as those occurring in *BTBD9* contribute to the exacerbation of RLS symptoms and whether these symptoms are ameliorated by rotigotine. We also injected wild-type (WT) and *Btbd9* mutant (MT) mice with serum from RLS patients with and without renal involvement who had and had not undergone hemodialysis to examine whether substances in RLS patient serum affect response of RLS symptoms to rotigotine.

## Results

### Confirmation of *Btbd9* mRNA expression in mutant mice brain

No mRNA expression of *Btbd9* was detected by reverse transcription polymerase chain reaction (RT-PCR) assay based on total RNA extracted from mice prefrontal cortex. On the other hand, WT mRNA expression of *Btbd9* was amplified obviously (Supplementary Fig. S1).

### Sleep at baseline and after sleep deprivation (SD)

**Figure 1:**
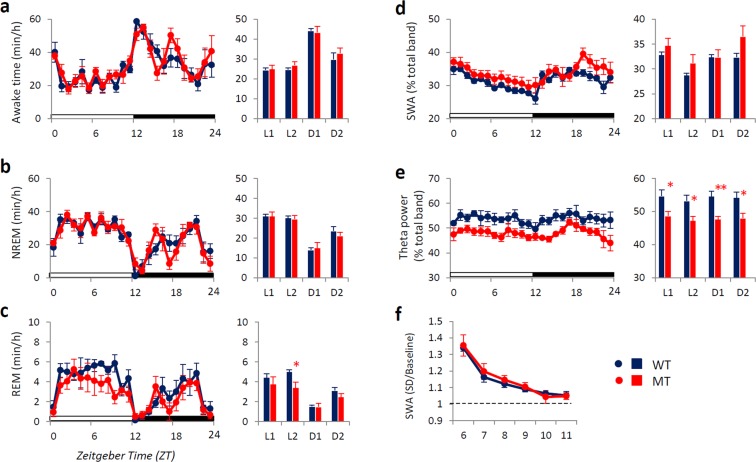
Sleep/wake patterns in *Btbd9* mutant (MT) mice at baseline. Hourly time course (left panels) and 6-hour bins (right panels; ZT0-6 (L1), ZT6-12 (L2), ZT12-18 (D1) and ZT18-24 (D2)) for (**a**) wakefulness; (**b**) Non-rapid eye movement (NREM) sleep; (**c**) Rapid eye movement (REM) sleep; (**d**) Slow-wave activity (SWA) in NREM sleep; (**e**) Theta power in REM sleep; (**f**) Rebound response of SWA after 6-hour sleep deprivation (SD); Blue circles and bars indicate wild-type (WT) mice, and red circles and bars indicate MT mice. All data are expressed as the means ± SEM (n = 6/group). *p < 0.05, WT versus MT mice. SEM, standard error of the mean.

The amounts of wakefulness and non-rapid eye movement (NREM) sleep observed for *Btbd9* MT mice were similar to those for WT mice (Fig. 1a,b), but the amount of rapid eye movement (REM) sleep was decreased compared with that of WT mice in the latter half of the light phase (Fig. 1c). MT mice showed increased slow-wave activity (SWA) in NREM sleep, and decreased electroencephalogram (EEG) theta power in REM sleep (Fig. 1d,e).

To evaluate sleep homeostasis, mice were sleep-deprived (SD) for 6 hours. During the recovery period after SD, both WT and MT mice showed increased SWA in NREM sleep (Fig. 1f).

### Baseline electromyogram (EMG)

**Figure 2:**
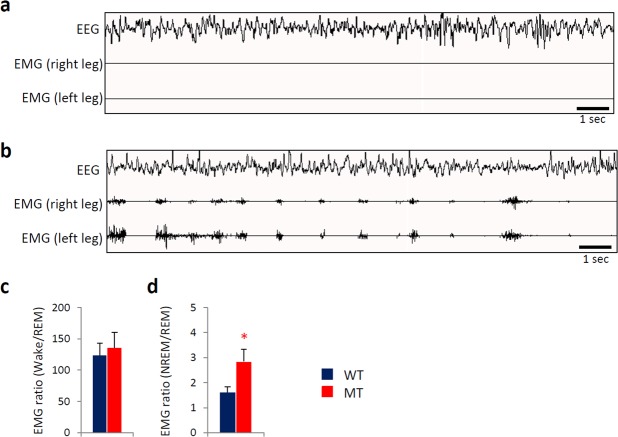
Leg muscle activity during non-rapid eye movement (NREM) sleep in *Btbd9* mutant (MT) mice at baseline. Consecutive waveform of representative EEG and EMG from gastrocnemius muscle isolated from the right and left legs of (**a**) wild-type (WT) and (**b**) MT mice. Integrated EMG power (averaged ZT0-6) during (**c**) wakefulness and (**d**) NREM sleep was normalized as a ratio to REM sleep in WT (blue bars) and MT (red bars) mice. All data are expressed as the means ± SEM (n = 6/group). *p < 0.05, WT versus MT mice. EEG, electroencephalogram; EMG, electromyogram; SEM, standard error of the mean.

In a comparison of representative waveforms of EEG/EMG for WT (Fig. 2a) and MT (Fig. 2b) mice, EMG data for gastrocnemius muscle from MT mice indicated increased leg muscle activity during NREM sleep compared with WT mice (Fig. 2d), whereas leg muscle activity during wakefulness was similar for the two genotypes (Fig. 2c).

### Effect of rotigotine on sleep in mice injected with serum from five RLS patients

**Figure 3:**
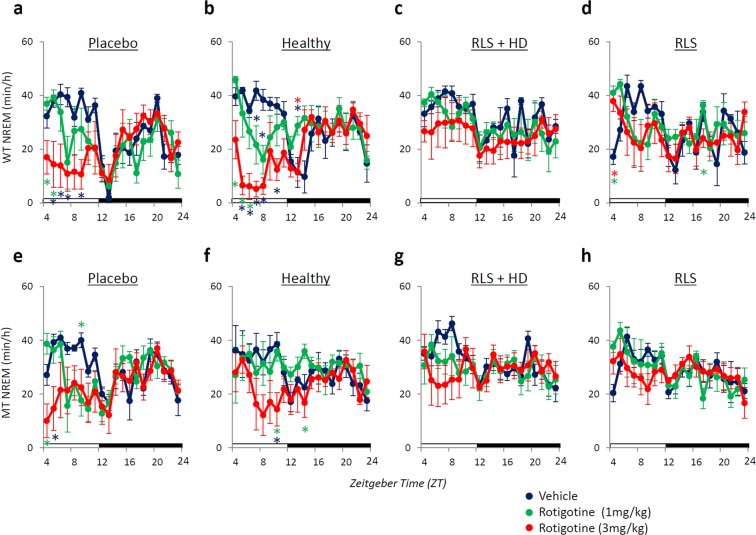
Effect of rotigotine on the amount of non-rapid eye movement (NREM) sleep in mice injected with RLS patient serum. Hourly time course for NREM sleep in wild-type (WT, **a**–**d**) and *Btbd9* mutant (MT, **e**–**h**) mice injected with (**a** and **e**) placebo, **(b** and **f**) serum from healthy subjects (Healthy), (**c** and **g**) serum from RLS patients with hemodialysis (RLS + HD), and (**d** and **h**) serum from RLS patients without hemodialysis (RLS). Blue, green, and red circles indicate vehicle, low dose of rotigotine, and high dose of rotigotine, respectively. All data are expressed as means ± SEM (n = 5/group). *(Blue) p < 0.05, versus vehicle; *(green) p < 0.05, versus low dose of rotigotine; *(red) p < 0.05, versus high dose of rotigotine. SEM, standard error of the mean.

**Figure 4:**
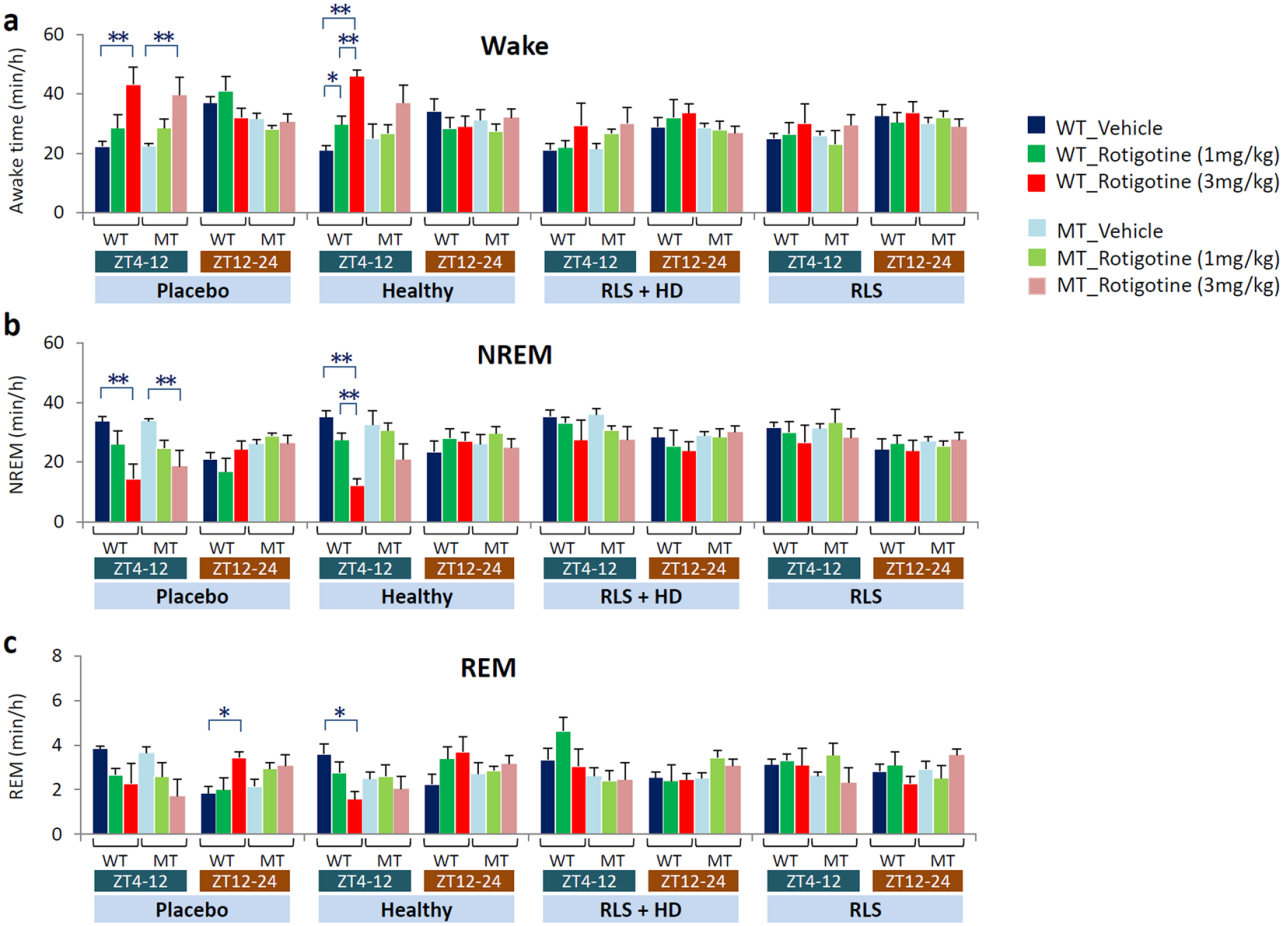
Sleep/wake patterns in mice injected with serum from RLS patient treated with rotigotine. Hourly averaged sleep/wake amounts in 8-hour periods of light (ZT4-12) and in 12-hour periods of dark (ZT12-24) phase for (**a**) wakefulness, (**b**) non-rapid eye movement (NREM) sleep, and (**c**) rapid eye movement (REM) sleep. Blue, green and red bars indicate mice injected with vehicle, low dose of rotigotine, and high dose of rotigotine, respectively. Dark shading represents wild-type (WT) and light shading indicates *Btbd9* mutant (MT) mice. All data are expressed as the means ± SEM (n = 6/group). *p < 0.05, **p < 0.01, among vehicle, low and high dose of rotigotine. SEM, standard error of the mean.

Next, we examined the effect of the dopamine agonist rotigotine (1 and 3 mg/kg, sc) on sleep and muscle activity in mice injected with serum from five RLS patients (Supplementary Table 1). Rotigotine treatment increased the amount of wakefulness and decreased the amount of NREM sleep in both WT and MT mice injected with placebo saline (Figs 3a,e and 4a,b; Supplementary Fig. S2a,e). In WT and MT mice injected with serum from healthy subjects, rotigotine treatment also increased the amount of wakefulness and decreased the amount NREM sleep, although the effect of rotigotine in MT mice was smaller than that for the WT mice (Figs 3b,f and 4a,b; Supplementary Fig. S2b,f). However, mice injected with serum from RLS patients who did or did not undergo hemodialysis did not exhibit a wakefulness-promoting effect of rotigotine (Figs 3c,d,g,h and 4a,b; Supplementary Fig. S2c,d,g,h).

**Figure 5:**
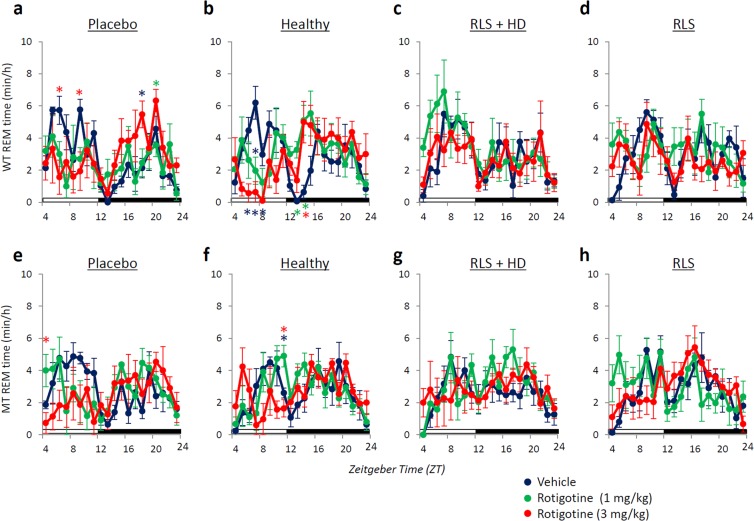
Effect of rotigotine on the amount of rapid eye movement (REM) sleep in mice injected with serum from RLS patients. Hourly time course for REM sleep in wild-type (WT, **a**–**d**) and *Btbd9* mutant (MT, **e**–**h**) mice injected with (**a** and **e**) placebo, (**b** and **f**) serum from healthy subjects (Healthy), (**c** and **g**) serum from RLS patients with hemodialysis (RLS + HD), and (**d** and **h**) serum from RLS patients without hemodialysis (RLS). Blue, green and red circles indicate vehicle, low dose of rotigotine, and high dose of rotigotine, respectively. All data are expressed as means ± SEM (n = 5/group). *(Blue) p < 0.05, versus vehicle; *(green) p < 0.05, versus low dose of rotigotine; *(red) p < 0.05, versus high dose of rotigotine. SEM, standard error of the mean.

The high-dose rotigotine treatment (3 mg/kg, sc) decreased the amount of REM sleep during the light phase in WT mice injected with placebo and serum from healthy subjects, and increased the amount of REM sleep during the dark phase (Figs 4c and 5a,b). In MT mice injected with placebo and serum from healthy subjects, low-dose rotigotine treatment (1 mg/kg, sc) slightly increased the amount of REM sleep during the light phase compared with vehicle and high-dose rotigotine (Fig. 4c and 5e,f). Meanwhile, rotigotine did not affect the amount of REM sleep in mice injected with serum from RLS patients with or without hemodialysis (Fig. 4c and 5c,d,g,h).

Dopamine agonists targeting the dopamine D_3_ receptor subtype have a higher efficacy on periodic leg movements and RLS than a drug that preferentially targets the D_2_ receptor subtype^20^. These results indicate that serum from an RLS patient diminished the wake-promoting effect of rotigotine treatment in mice. However, the influence of dopamine D_2_/D_3_ agonist to sleep structure, human studies will be necessary in the future.

### Effect of rotigotine on muscle activity during NREM sleep in mice injected with serum from five RLS patients

**Figure 6:**
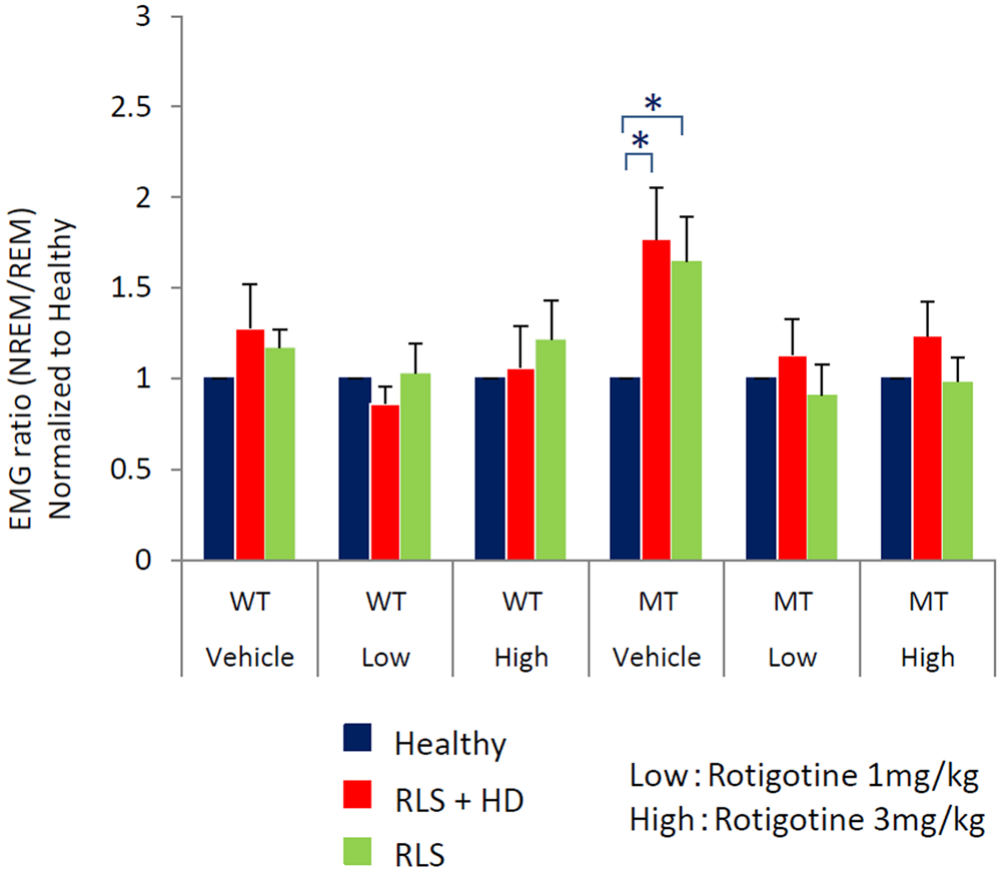
Effect of rotigotine on leg muscle activity during non-rapid eye movement (NREM) sleep in mice injected with serum from RLS patients. Normalized EMG in NREM sleep was averaged over 24 hours for wild-type (WT) and *Btbd9* mutant (MT) mice treated with rotigotine after serum injections. Blue, red, and green bars indicate serum from healthy subjects (Healthy), RLS patients with hemodialysis (RLS + HD), and RLS patients without hemodialysis (RLS), respectively. All data are expressed as the means ± SEM (n = 5/group). *p < 0.05 versus healthy. SEM, standard error of the mean.

Leg movement measured by EMG during NREM sleep was increased in MT mice injected with serum from RLS patients with or without hemodialysis (Fig. 6). In mice treated with rotigotine after serum injection, the amount of leg movement decreased for mice treated with serum from patients who either did or did not undergo hemodialysis. These results suggest that serum from five RLS patients can increase leg muscle activity during NREM sleep in MT mice, and that rotigotine treatment ameliorates this effect.

## Discussion

To our knowledge, this is the first study to measure changes in sleep conditions and periodic limb movements in sleep (PLMS) in an animal model of RLS in a related genetic background after administration of serum from a patient with severe chronic kidney disease and to assess the effects of a dopamine-receptor agonist on PLMS in these animals. In baseline sleep recordings, the amounts of wakefulness and NREM sleep of MT and WT mice were similar, whereas the amount of REM sleep was decreased for MT mice relative to WT mice. Reportedly, RLS patients show a reduction in REM sleep duration and increase in REM sleep latency^21^. Moreover, the amount of slow wave sleep for RLS patients was similar to that for healthy controls, but NREM sleep duration was also reduced^21^. Thus, our results were partially consistent with earlier studies in that we observed decreased amounts of REM sleep in the RLS model, yet saw similar basal amounts of NREM sleep for MT and WT mice. Meanwhile, basal EMG data for leg muscle showed that MT mice exhibited increased leg muscle activity during NREM sleep compared with WT mice. These results support published previously studies that reported a strong linkage between *BTBD9* and increased PLMS^2,22^.

Pramipexole, a dopamine D_2_/D_3_ receptor agonist, is frequently used to treat RLS and has a sleep disrupting effect in mice^23^. In rats, systemic injection of a D_2_ receptor agonist induces biphasic effects, wherein low and high doses reduce and increase wakefulness, respectively^24^. Here we found that a high-dose rotigotine treatment indeed increased the amount of wakefulness in both WT and MT mice injected with placebo and serum from healthy controls. However, this wake-promoting effect of rotigotine was not observed in mice injected with serum from RLS patients who did or did not undergo hemodialysis. Although the explanation for why a wake-promoting effect of rotigotine was induced only in mice injected with placebo and healthy control serum is unclear, our results suggest that serum from patients with idiopathic and renal RLS may contain substances that counteract the arousal effect of rotigotine. These substances may be a factor in the increased muscle activity of RLS patients, regardless of the presence of uremic substances. In addition, genotype also contributed to these differences in that WT mice tended to show more conspicuous effects of rotigotine compared to MT mice, suggesting that the presence or absence of *Btbd9* gene mutations may also be involved in the wake-promoting effect of rotigotine. Such mutations could be associated with RLS pathophysiology, a possibility that requires further exploration.

We considered that an increase in muscle activity during sleep in MT mice corresponds with PLMS, a major RLS symptom. PLMS is also associated with other diseases, but PLMS with and without RLS was significantly related to *BTBD9* polymorphisms^2,22^. Here we confirmed that the PLMS findings in the EMG of MT mice were similar to those of the putative rat RLS model^14^.

In EMG taken during NREM sleep, we observed increased leg movement in MT mice injected with serum from RLS patients who either had or had not undergone hemodialysis. Rotigotine treatment ameliorated this effect, suggesting that the injected serum could contain substances that induced increased muscle activity and that the dopaminergic system mediates these effects. Future studies will be needed to detect and identify such substances, which could guide the development of novel therapeutics for RLS.

In conclusion, we found that administration of serum from RLS patients increased muscle activity during NREM sleep in MT mice with disrupted Btbd9, and this increase in muscle activity was suppressed by rotigotine treatment. We also observed that serum from RLS patients could counteract the arousal effect of rotigotine treatment in mice. Our data strongly support previously published reports showing that *BTBD9* and the dopaminergic system are linked to RLS, and partially clarify the pathophysiological mechanisms of RLS, which may inform new approaches for treating RLS.

## Materials and Methods

### Animals

MT mice were prepared at Gunma University. Mice were kept under pathogen-free barrier conditions, and animal procedures were performed in accordance with institutional and national regulations following approval by The Gunma University Animal Care and Experimentation Committee. The commercial embryonic stem (ES) cell line (RRE078, BayGenomics) containing a gene trap vector in intron 6 of the *Btbd9* gene were obtained from the Mutant Mouse Resource & Research Center (MMRRC, UC Davis, CA, USA) and injected into blastocysts. These mice were identical to other mice previously described^25^. Chimeric mice were crossed to C57BL/6 mice. Heterozygous mice (Btbd9^+/−^ mice) were further backcrossed to C57BL/6 mice for at least eight generations and the resulting heterozygous animals were crossed to produce homozygous mice. *Btbd9* MT mice were then transferred from Gunma University to Tokushima University for experiments. PCR was used for identifying the wild-type heterozygous-type and homozygous-type alleles for genotyping. When we used BtbGenome primers 1 (Btb F: TTCCTGGTCCTCCTGCTACC) and 2 (Btb R: ACCCGTGGGAAAGCTTAGTG) for identifying the 900 bp wild-type allele. To confirm the presence of the 450 bp trap allele, we used Btb F and trap vector primers 3 (Trap R: CCACAACGGGTTCTTCTGTT). We used homozygous mice for all experiments.

The mRNA expression of Btbd9 was confirmed by reverse transcription polymerase chain reaction (RT-PCR). Total RNA was purified from mouse cerebrum using Trizol reagent (Invitrogen, Carlsbad, CA, USA), followed by DNase digestion and purification using the RNeasy kit (QIAGEN, Valencia, CA, USA). cDNA synthesis was performed on total RNA extracted from the mice prefrontal cortex using SuperScript™ VILO™ cDNA Synthesis Kit (Thermo Fisher Scientific, Waltham, MA, USA). Then PCR was performed with two primer sets, the forward primer located in the exon 4 (Exon5-6F – 5′- AGCTTCTGAACGTCGTGAGG-3′)and reverse primer in the exon 5 before the intron containing the gene-trap (Exon5-6R – 5′- TCATCATCAATGGGGTGCCG-3′), the others forward primer located in the junction of exon 6 before the intron containing the gene-trap and exon7 after this intron (Exon6-7F – 5′-GCCCGTGTCTGCAGGTATAT-3′) and the reverse primer located in the exon 10 (Exon10R – 5′-TTCGGATGAAGGATGCAGGC-3′). (Supplementary Fig. S1).

Seven-month-old male MT mice and their wild-type littermates were used in this study. Food and water were available *ad libitum*. A 24-hour light-dark cycle (lights on for 12 hours, off for 12 hours) was maintained throughout the study (lights on at zeitgeber time [ZT] = 0 at 08:00 am). Room temperature was maintained at 23 ± 1 °C throughout the experimental period. The study protocol received approval from the Gunma University Gene Recombination Experiment Safety Committee and the Animal Experimentation Committee of Tokushima University. All experiments involving mice were performed in accordance with Guidelines for the Care and Use of Animals approved by the Council of the Physiological Society of Japan.

### Sleep recordings and analysis

Mice were anesthetized with a cocktail of ketamine (100 mg/kg) and xylazine (25 mg/kg) for EEG/EMG implantation surgery. Stainless steel miniature screw electrodes were implanted in the skull of the mice to record EEGs, and Teflon-coated stainless steel wires were implanted in the gastrocnemius muscle of both legs to record EMGs. After a 2-week recovery period, the mice were transferred to plastic cages (20 × 24 × 30 cm) in a sound-proof recording room and allowed to habituate for two days. The mice were connected by flexible cables to a polygraph and computer-assisted data acquisition system. EEG/EMG signals were captured at 128/500 Hz using a sleep recording system (Vital Recorder, Kissei Comtec, Matsumoto, Japan). Mice were also monitored with a video recording system.

Off-line sleep scoring was performed by visual assessment of EEG and EMG activities using the SleepSign analysis program (Kissei Comtec, Matsumoto, Japan). Vigilance states were classified for each 10-second period as wakefulness, REM sleep, or NREM sleep. The EEG and EMG power spectrum in the period that was scored as NREM sleep was calculated by Fast Fourier Transform using the SleepSign analysis program. The EEG delta and theta frequency bands were set at 0.5–4.0 Hz (SWA) and 4.5–10.5 Hz, respectively. The delta and theta power were normalized and described as a percentage of the total power (0.5–30 Hz), and the data were averaged at hourly intervals. The EMG total power (50–250 Hz) in each vigilance stage was integrated and normalized as a ratio to that of REM sleep. The EEG/EMG data in periods including artificial/electrical noise were excluded from analysis after checking the video recording and movement of the mice.

### Sleep deprivation

To evaluate sleep homeostasis, mice were sleep-deprived (SD) for 6 hours, between ZT0 and ZT6, using a small, soft brush to touch the backs of mice that appeared to be somnolent. At ZT6, SD was terminated, and the EEG and EMG were recorded for an 18-hour period of uninterrupted recovery sleep.

### Human serum preparation

Serum from 5 healthy subjects and 5 patients with idiopathic RLS and 5 RLS patients with secondary to end-stage renal disease was filtered using a Minisart filter (pore size, 0.2 μm; Sartorius AG, Göttingen, Germany). After filtration, identical volumes of serum from each subject in the same group (healthy subjects, idiopathic RLS patients and patients with RLS and secondary to end-stage renal disease) were mixed and the mixtures were used for intraperitoneal (ip) injection into the mice. All 5 RLS patients with secondary to end-stage renal disease were received hemodialysis for 4 hours three times a week. The serum was obtained from the different patients before hemodialysis. The administration of dopamine agonist was withdrawn for seven days. This study was conducted in accordance with the Declaration of Helsinki. Informed consents for participating this study had obtained from all participants. Research protocol and informed consent were approved by Medical Research Ethics Committee of Tokyo Medical University. All experiments were performed in accordance with the Ethical Guidelines for Medical and Health Research Involving Human Subjects in Japan.

### Pharmacological treatments and injection

Four hours after ip injection with human serum, rotigotine was subcutaneously (sc) administered to the mice. Human serum (0.5 ml/30 g mouse; 166.7 ml/kg, ip) or placebo (saline) was administered to each animal at ZT0. Rotigotine (1 and 3 mg/kg dissolved in olive oil, Sigma-Aldrich Japan, Tokyo, Japan, sc) was administered to each animal at ZT4 (Supplementary Table 1). Sleep data for the 16 hours following serum administration were captured and analyzed. Each drug/serum was administered in a random order with a washout period of 1 week.

### Statistics

Results are expressed as the mean ± SEM. The data in Figs 1–5 are normally distributed, while the data in Fig. 6 is not normally distributed (tested by Kolmogorov-Smirnov test). The data were analyzed by repeated measurement of two-way ANOVA for the hourly time course and the Student’s t-test for comparison between two groups in each bin (Fig. 1), Student’s t-test (Fig. 2), repeated measurement of two-way ANOVA followed by a Bonferroni/Dunn post-hoc test (Figs 3 and 5), one-way ANOVA followed by a Bonferroni/Dunn post-hoc test (Fig. 4), and Kruskal-Wallis test followed by Willcoxon’s test (Fig. 6). For all comparisons, the criterion for significance was p < 0.05 (2-tailed).

## Supplementary information

Supplementary information
