# Supplementary material for: Rotigotine suppresses sleep-related muscle activity augmented by injection of dialysis patients’ sera in a mouse model of restless legs syndrome
Source: Sci Rep. 2019 Nov 8;9:16344. doi: 10.1038/s41598-019-52735-z (PMC6841937; doi:10.1038/s41598-019-52735-z)
Supplement: Supplementary file 1 — Supplementary information [file 41598_2019_52735_MOESM1_ESM.pdf]

Supplementary information

Rotigotine suppresses sleep-related muscle activity augmented by injection of dialysis patients' sera in a mouse model of restless legs syndrome

Kazuhiro Muramatsu[1,2*], Sachiko Chikahisa[3*], Noriyuki Shimizu[3], Hiroyoshi Séi[3], Yuichi Inoue[4†]

[1]Department of Pediatrics, Jichi Medical University, Tochigi, Japan.

[2]Department of Pediatrics, Gunma University Graduate School of Medicine, Gunma, Japan.

[3]Department of Integrative Physiology, Institute of Biomedical Sciences, Tokushima University Graduate School, Tokushima, Japan.

[4]Department of Somnology, Tokyo Medical University, Tokyo, Japan.

[*]These authors contributed equally to this work.

[†]Corresponding author: Yuichi Inoue, M.D., Ph.D.

Department of Somnology, Tokyo Medical University, 6-7-1 Nishishinjuku, Shinjuku-ku, Tokyo 160- 0023, Japan.

E-mail: inoue@somnology.com, Tel.: +81-3-3460-3033, Fax: +81-3-3460-3033

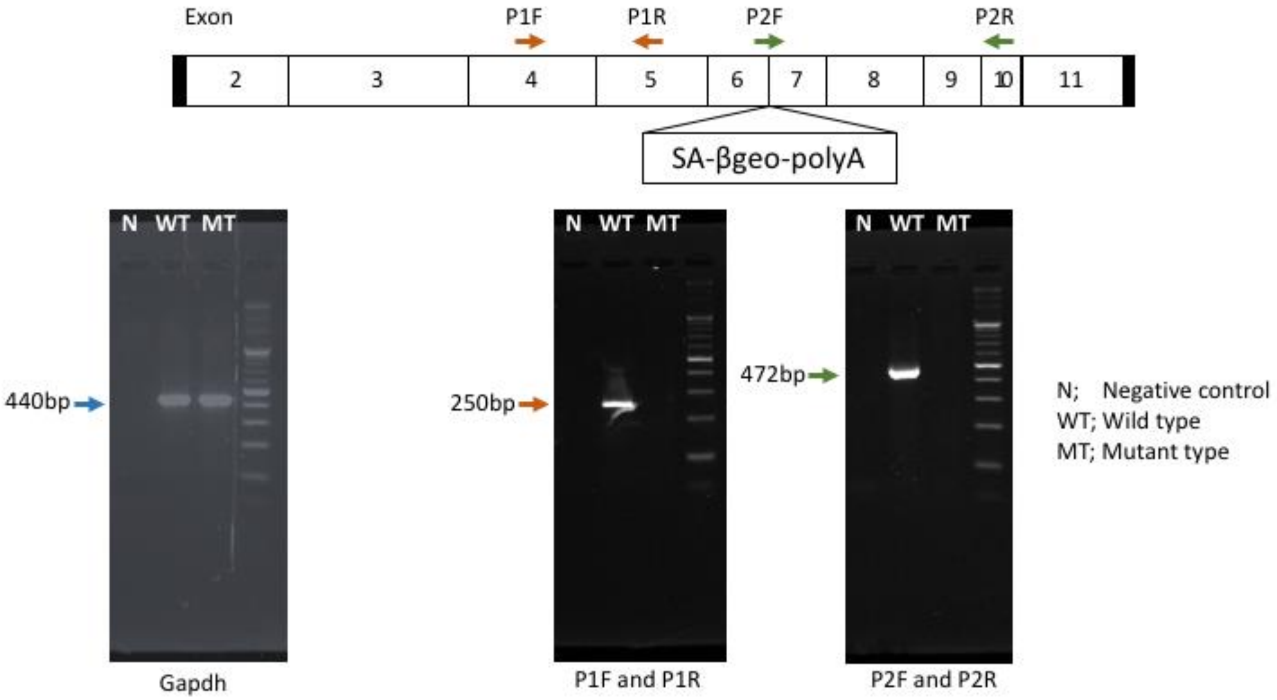

Supplemental figure S1. Confirmation of mRNA deletion in Btbd9 mutant mice brain.

The wild-type (WT) allele of the Btbd9 gene contains 12 exons. A gene trap allele of the Btbd9 gene has a splice acceptor (SA), β-geo, and polyA signal (polyA) inserted into between exon 6 and 7. Open vertical rectangles represent coding exons. Primer location are indicated on this schema. P1F; Exon5-6F, P1R; Exon5-6R, P2F; Exon6-7F, P2R; Exon10R. The WT mice, produced both Btbd9 gene intact fragment existing before gene-trap and over it in RT-PCR. In contrast no fragments were amplified in the homozygous Btbd9 mutant mice. Glyceraldehyde-3-phosphate dehydrogenase (Gapdh) was amplified as internal control. This PCR was used these primers set of forward (Gapdh-F: CGGGGCCCACTTGAAGG) and reverse (Gapdh-R: TACTTGGCAGGTTTCTCCAGG) for 440bp fragment.

Supplemental Table 1. Experimental groupings and conditions. Wild-type (WT) and *Btbd9* mutant (MT) mice received one of the following treatments: saline (placebo); serum from healthy subjects (Healthy) serum from RLS patients with (RLS + HD); or serum from RLS patients without hemodialysis (RLS). Under each condition, olive oil (vehicle), low dose of rotigotine (Low) or high dose of rotigotine (High) were administered as indicated.

Serum \ Rotigotine	Olive oil (Vehicle)	Rotigotine (Low dose)	Rotigotine (High dose)
Saline (Placebo)	Vehicle	Low (1 mg/kg)	High (3 mg/kg)
Healthy subjects (Healthy)	Vehicle	Low (1 mg/kg)	High (3 mg/kg)
RLS with hemodialysis (RLS + HD)	Vehicle	Low (1 mg/kg)	High (3 mg/kg)
RLS without hemodyaliysis (RLS)	Vehicle	Low (1 mg/kg)	High (3 mg/kg)

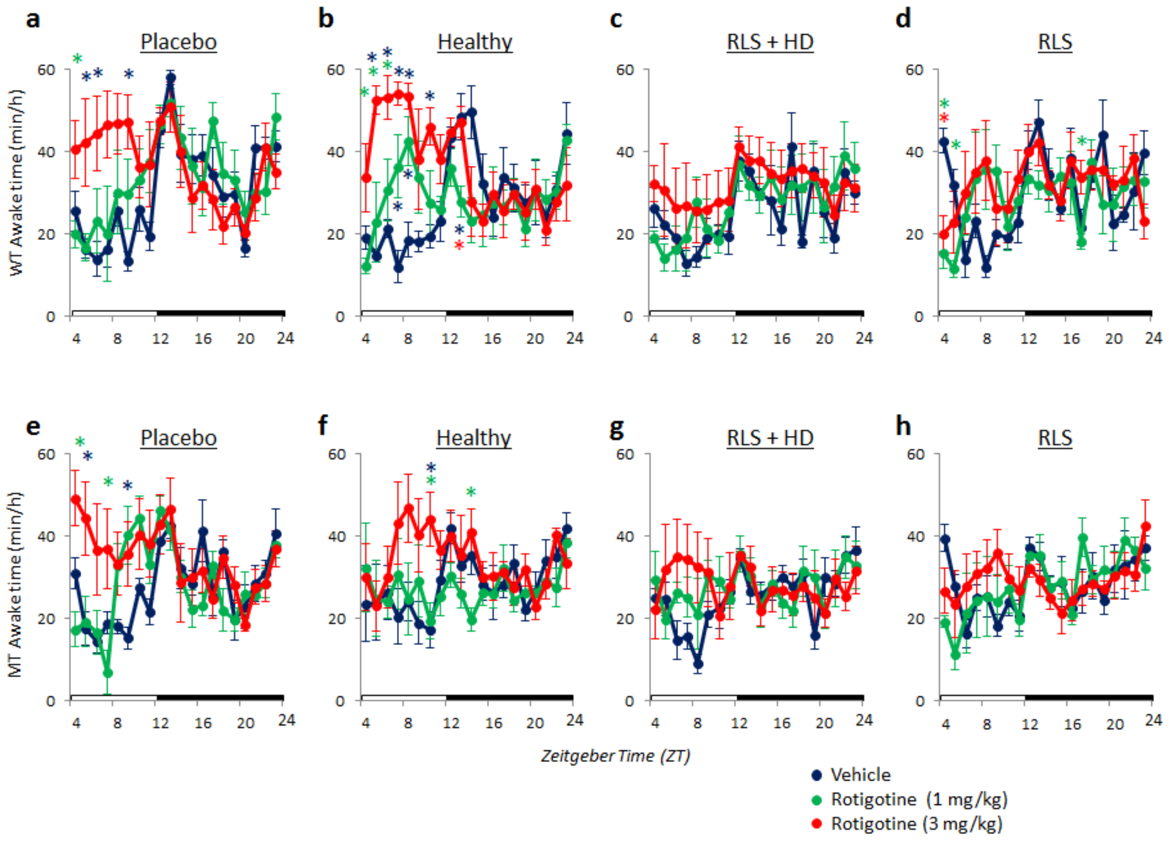

Supplemental figure S2. Effect of rotigotine on the amount of the wakefulness period in mice injected with serum from RLS patients.

Hourly time course for wakefulness in wild-type (WT, a-d) and Btbd9 mutant (MT, e-h) mice injected with (a and e) placebo, (b and f) serum from healthy subjects (Healthy), (c and g) serum from RLS patients with hemodialysis (RLS + HD), and (d and h) serum from RLS patients without hemodialysis (RLS). Blue, green and red circles indicate vehicle, low dose of rotigotine, and high dose of rotigotine, respectively. All data are expressed as means ± SEM (n = 5/group). *(blue) $p < 0.05$, versus vehicle; *(green) $p < 0.05$, versus low dose of rotigotine; *(red) $p < 0.05$, versus high dose of rotigotine. SEM, standard error of the mean.
